# Individual Placement and Support (IPS) beyond severe mental health: An overview review and meta-analysis of evidence around vocational outcomes

**DOI:** 10.1016/j.pmedr.2024.102786

**Published:** 2024-06-10

**Authors:** Adam Whitworth, Susan Baxter, Jane Cullingworth, Mark Clowes

**Affiliations:** a Strathclyde Business School, University of Strathclyde, 199 Cathedral St, Glasgow G40QU, UK; b University of Sheffield, School for Health and Related Research, Regent Court, Regent Street, Sheffield S14DA, UK; c School of Social and Political Sciences, Adam Smith Building, University of Glasgow, G128RT, UK

**Keywords:** Individual placement and support, IPS, Fidelity, Vocational outcomes, Mental health

## Abstract

**Objective:**

To provide an overview review of international evidence of vocational outcomes in Individual Placement and Support (IPS) interventions for populations other than severe mental health.

**Methods:**

An overview of reviews published in English since 2000 reporting vocational outcomes (job entry, work sustainment, earnings, work hours, time to job entry) against counterfactuals of IPS interventions for population groups other than severe mental health. The overview review maximises data from individual studies and includes additional recent primary studies. DerSimonian-Laird random effects meta-analysis was performed.

**Results:**

Thirteen eligible studies were identified from five reviews and five more recent individual studies were also identified. IPS studies covered a range of groups with a concentration towards mental health. For the primary vocational outcome of job entry all IPS studies showed superior job entry rates compared to control groups with an overall weighted odds ratio of 1.78 [1.42,2.22]. Substantial heterogeneity was identified by study size and the overall weighted odds ratio of 1.32 [1.2,1.46] estimated from the large and medium sized studies seems a more plausible estimate of the likely effects of scaled-up IPS interventions in groups beyond severe mental health. Secondary vocational outcomes including job sustainment, total earnings, average weekly hours worked and time to job entry were typically superior in IPS services than control groups.

**Conclusions:**

IPS services are consistently more effective in supporting diverse population groups into sustained employment compared to business-as-usual employment services. The evidence is limited by unclear terminology, small sample sizes, incomplete intervention fidelity, intervention contamination and inconsistent measurement.

## 1 Introduction

### 1.1 Rationale

One in seven working age adults across OECD countries identify as having a disability (OECD, 2022) and the disability employment gap – the difference in the percentage of working age adults in employment with and without a disability – remains close to 30 % points as an OECD average (OECD, 2022). This is despite a substantial proportion of non-working disabled people stating that they wish to work given the right job and support (Employment of disabled people, 2022) and despite evidence that wider social determinants – with employment key amongst them – account for a far larger share of the variation in people’s health outcomes than clinical care (Braveman and Gottlieb, 2014).

Individual Placement and Support (IPS) is a distinctive Supported Employment model of voluntary employment support for workless individuals with health conditions and disabilities. IPS was created in the 1990 s in the USA to offer employment support to people with severe mental health conditions. IPS services adopt a place-then-train approach of rapid job search and entry into paid work in the open labour market whilst simultaneously supporting health (and other) support needs. This contrasts with standard approaches that seek to first tackle barriers before next considering job search and entry (i.e. a train-then-place approach) and/or that consider either unpaid voluntary work or sheltered employment as successful outcomes.

IPS services are distinctive in their adherence to a fidelity scale (The IPS fidelity scale [Internet] Centre for Mental Health, 2023) that provides a list of key service characteristics evidenced to associate with positive outcomes (Winter et al., 2020, Yamaguchi et al., 2022). Two key fidelity items inside IPS services are integration of IPS employment specialists inside clinical teams (e.g. mental health teams) to offer joined-up support as well as proactive employer engagement in order to support transitions into jobs that are well matched to client preferences and needs. Clients in IPS services receive personalised support framed around five key phases – engaging and referring clients, vocational profiling, proactive employer engagement, job matching and securing employment, and in-work support. Across that support IPS services show a set of progressive, person-centred values – voluntary participation, strengths-based support, client preferences and agency, co-production, and intensive and personalised support.

IPS services are well evidenced to be effective in their traditional severe mental health population group with around 30 randomised controlled trials (RCTs) showing average job entry rates of 55 % in IPS interventions compared to 25 % in control groups (The evidence for IPS, 2023, Bond et al., 2020). As such, IPS has become the dominant evidence-based employment model internationally for people with severe mental health issues.

As a result of these impacts, IPS has also become the subject of fast-moving and varied experimentation and trialling in wider healthcare settings and population groups including common mental health, musculoskeletal issues, chronic pain, substance misuse, spinal cord injury, trauma, homelessness and young people. Reviewing this rapidly evolving and fragmented IPS beyond severe mental health evidence base is a priority. As detailed below, a small number of reviews have already been conducted which offer helpful reference points into this rapidly evolving evidence landscape. However, existing reviews are partial and inconsistent in their identified studies, incomplete in their reporting and already dated.

### 1.2 Objectives

In response, the present article offers a consolidated, comprehensive and current review of the literature and evidence around the vocational impacts of IPS services beyond severe mental health. To do so the article provides a comparative understanding of existing reviews, a consolidated presentation of all findings within reviews and underlying individual studies, an updating with more recent studies, and a meta-analysis of weighted overall effects across individual studies.

## 2 Methods

### 2.1 Eligibility criteria

The present overview review forms part of a wider systematic review. We follow PRISMA guidelines (Page et al., 2020) for the reporting of reviews and PRIOR guidelines (Gates et al., 2022) for the reporting of overview reviews.

The eligibility criteria for the underlying systematic review are:

• Population: any population group other than severe mental health. Where studies included both those with and without severe mental health the study was deemed eligible if more than half of the study participants did not have severe mental health;
• Intervention: employment interventions that follow IPS fidelity or the overarching five-phase Supported Employment approach;
• Comparison: no counterfactual is required and qualitative data are included;
• Outcomes: any vocational (i.e. employment related) outcomes reported (e.g. job entry, job sustainment, hours worked, income, time to job entry).
• Time and geography: studies published in English since 2000 from high-income or upper-middle-income nations as defined by the World Bank Atlas method since these dominate application of IPS interventions and provide similarity in economic and welfare contexts.

Once eligible review studies were identified for this overview review, individual studies inside those reviews were eligible for the present analyses where they satisfied all of the above eligibility criteria excepting regards comparison where individual studies were additionally required to have a sufficiently robust counterfactual given the present focus is robust impact evidence around vocational outcomes.

### 2.2 Information source, search strategy and ethics

An information specialist (MC) developed a bespoke search strategy and ran searches in MEDLINE, PsycINFO, CINAHL, Social Sciences Citation Index via Web of Science and ProQuest Social Science Collection electronic databases in April 2022. Reference lists of studies identified as potentially relevant were scrutinised, known systematic reviews were followed, key websites assessed (Centre for Mental Health, IPS Grow, British Association for Supported Employment, Department for Work and Pensions) and key networks (experts, commissioners and providers) were consulted. To ensure that our included literature was up to date we conducted further forward citation searches from the identified review studies in January 2024. We identified five additional individual studies and no further reviews that met our study eligibility criteria.Search keywords and an example search strategy are included in the supplementary online material. All data were public domain and the research received ethical approval from the lead author’s (AW) institution.

### 2.3 Selection process

Retrieved citations were added to an Endnote database for systematic screening by two members of the team, one performing initial screening (JC) and the other second screening and managing the search and screening work (SB). Differences in decision were recorded and discussed between the reviewers and the study lead (AW) to reach consensus. Citations were screened initially at title and abstract level with those of potential relevance tagged and sourced as full text. Studies that met the eligibility criteria after full text scrutiny were included. Five reviews were identified as meeting the eligibility criteria for this overview containing 13 eligible individual studies. Individual studies inside these reviews not eligible for our overview review are detailed in the supplementary online materials including reason for exclusion. A further 5 very recent eligible studies were identified from the follow-on citation searches of the reviews.

### 2.4 Data collection process

Reviews varied in the completeness of their reporting of individual study data. For the present overview review all vocational data were extracted from reviews and individual studies to maximise comprehensiveness. Relevant information was captured in Excel tables.

### 2.5 Data items and risk of bias assessment

Data were sought for any outcome variable relating to paid employment outcomes (e.g. job entry, time to job entry, job sustainment, pay, hours worked, etc) for both IPS services and their control groups. The primary outcome is job entry rate measured as the percentage of the service (IPS or business-as-usual) caseload that successfully moves into paid employment during/at a specific period/point. Data were sought on study country, population group(s) supported, sample sizes, IPS intervention details, fidelity scores, nature of the control group and impact evaluation methodology. Risk of bias of the individual studies was assessed using the revised Cochrane risk-of-bias-tool (RoB2).

### 2.6 Synthesis methods

Data are synthesised in graphical and tabular form. Given variability in how data items are measured across studies data definitions are provided where needed. To estimate the overall effect size on the primary outcome random effects meta-analysis was performed and a forest plot created using the **metan** command in Stata 18 with a DerSimonian-Laird estimate of the between-study variance. The primary outcome is the odds ratio of job entry rates between IPS and control services (where odds ratios greater than one favour IPS). Random-effect *meta*-analysis is appropriate given the expectation from existing review studies of heterogeneity across studies due to their differing population groups, contexts, interventions and sample sizes. Heterogeneity is measured using the I^2^ statistic. I^2^ values exceeding 50 % indicate substantial heterogeneity and I^2^ values less than 30 % indicate low heterogeneity. Funnel plots are used to sensitivity test the overall effect to small-study effects and potential publication bias.

### 2.7 Reporting bias and certainty assessment

Risk of reporting bias is low. Maximum possible data were extracted from individual studies as well as review studies. Missing data were minimal and are shown as missing where relevant. No imputation of missing data was conducted. The GRADE framework was used to assess certainty over five dimensions: study limitation, inconsistency, indirectness, imprecision and publication bias.

## 3 Results

### 3.1 Systematic review and supplemental primary study selection

**Fig. 1:**
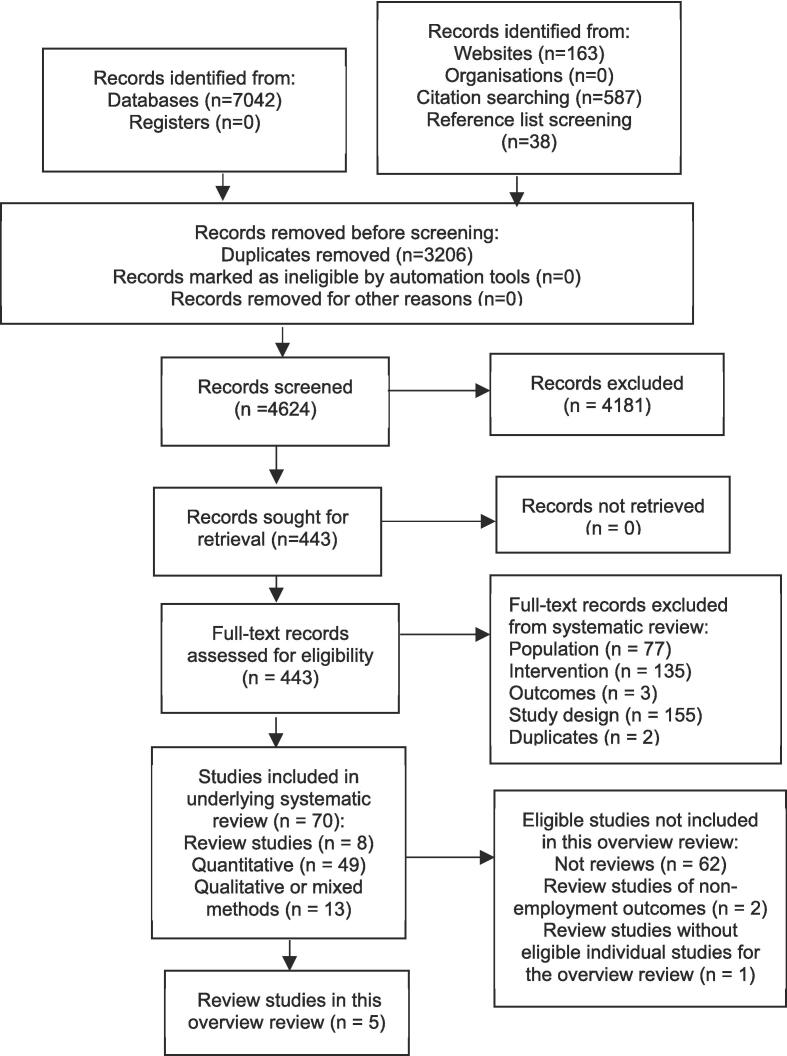
PRISMA diagram.

Fig. 1 shows the selection process. After duplicates were removed 4,624 records were identified for screening from which 5 eligible reviews were identified.

### 3.2 Primary study overlap

The columns of Table 1 show the 5 review studies identified (Probyn et al., 2021, Taylor et al., 2012, Bond et al., 2019, Jetha et al., 2019, Fadyl et al., 2020, Harrison et al., 2020), rows show the differing population groups in the individual studies, and table cells list the 18 eligible individual studies. A final column details the 5 additional recent studies identified subsequent to initial searches and to these reviews (Davis et al., 2022, Sveinsdottir et al., 2022, Newton et al., 2023, Brinchmann et al., 2024, Marsden et al., 2024).

**Table 1:** Distribution of studies across reviews and population groups.

	Bond et al. (2019)	Fadyl et al. (2020)	Probyn et al. (2021)	Harrison et al. (2020)	Jetha et al. (2019)	Additional recent studies
**Review Focus/Population Group**	**RCTs of IPS beyond SMI**	**RCTs of vocational interventions for mild to moderate mental health**	**RCTs of SE beyond SMI**	**Studies of IPS for substance misuse**	**Studies of vocational interventions for young adults with chronic health conditions**	
**Common mental Health (CMH)**	Reme et al., 2015, Hellstrom et al., 2017	Reme et al., 2015, Hellstrom et al., 2017				Davis et al. (2022)
**CMH &/or musculoskeletal (MSK)**						Newton et al. (2023)
**CMH or somatic disorder**						Brinchmann et al. (2024)
**Moderate to severe mental health**	Reme et al. (2019)	Reme et al., 2019, Poremski et al., 2017	Poremski et al. (2017)		Ferguson et al. (2012)	
**Affective disorder**	Bejerholm et al. (2017)	Bejerholm et al. (2017)	Bejerholm et al. (2017)			
**PTSD veterans**	Davis et al., 2012, Davis et al., 2018	Davis et al., 2012, Davis et al., 2018	Davis et al. (2012)			
**Substance misuse**	Lones et al., 2017, LePage et al., 2016 67.		Lones et al., 2017, LePage et al., 2016 67.	Lones et al., 2017, LePage et al., 2016 67., Rosenheck and Mares, 2007		Marsden et al. (2024)
**Spinal cord injury**	Ottomanelli et al. (2017)		Ottomanelli et al. (2017)			
**NEET young adults**			Sveinsdottir et al. (2020)			
**Chronic pain**						Sveinsdottir et al. (2022)

### 3.3 Characteristics of systematic reviews and supplemental primary studies

Table 2 provides details of the 18 individual IPS studies. The studies cover a diverse range of groups although with a concentration towards low to moderate mental health (sometimes in combination with other conditions). Thus, whilst IPS innovation beyond its traditional severe mental health group has occurred its use still remains focused to a significant degree on mental health. The evidence is concentrated in the USA (10 studies) and Scandinavia (4 Norwegian studies, 1 Swedish and 1 Danish) and with 2 large recent UK trials. Four studies report on significantly modified forms of IPS (Rosenheck and Mares, 2007, LePage et al., 2016 67., Hellstrom et al., 2017, Sveinsdottir et al., 2020) that omit core aspects of the model whilst three studies report on interventions that combine IPS with other interventions (Reme et al., 2015, Poremski et al., 2017, Bejerholm et al., 2017). In terms of evaluation design, randomised controlled trials (RCTs) are widespread. Two studies show weaker impact evaluation designs and offer more questionable counterfactuals (Rosenheck and Mares, 2007, Ferguson et al., 2012).

**Table 2:** Characteristics of included studies.

**Study**	**Broad Condition/ Population group**	**Condition/ Population details**	**Country**	**Sample Size (t)**	**Sample size (c)**	**Intervention**	**IPS Fidelity Level**	**Control Group**	**Method**
Reme et al. (2015)	Common mental health	Sick leave, at risk of sick leave, long-term disability benefits	Norway	630	563	IPS + work-focused CBT	Not reported	List of job resources	RCT
Hellstrom et al. (2017)	Common mental health	Mood and anxiety disorders	Denmark	162	164	Modified IPS (no integration, benefits counselling & minimal job development)	Not reported but likely low	Non-integrated BAU services	RCT
Davis et al. (2022)	Common mental health	Veterans with non-psychotic mental health and primary care link	US	58	61	IPS	Fair improved to good	Standard vocational rehabilitation	RCT
Newton et al. (2023)	Common mental health &/or physical health	Common mental health and/or physical health in primary care	UK	4,896	4,889	IPS	Fair to good	Standard vocational rehabilitation	RCT
Davis et al. (2012)	Mild to moderate mental health	Veterans with PTSD	US	42	43	IPS	Fair	Compensated work therapy	RCT
Davis et al. (2018)	Mild to moderate mental health	Veterans with PTSD	US	271	270	IPS	Fair improving to good	Compensated work therapy	RCT
Reme et al. (2019)	Moderate to severe mental health	Depression, psychosis, panic disorders, drug/alcohol, bipolar disorder, anxiety	US	229	181	IPS	Below fair improved to fair to excellent	Non-integrated BAU services	Multi-site RCT (unbalanced groups)
Poremski et al. (2017)	Moderate to severe mental health + housing issues	Homeless or precariously housed 18–24 yr olds with major depression, mania or hypomania, PTSD, panic disorder, mood disorder with psychotic features, psychotic disorder	US	44	41	IPS + Housing First	Fair improving to good	Non-integrated BAU services	Stratified RCT
Ferguson et al. (2012)	Moderate to severe mental health + housing issues	Homeless 18–24 yr olds with major depression, generalized anxiety, mania or hypomania, antisocial personality disorder, PTSD, substance misuse	US	20	16	IPS	Not reported	Non-integrated BAU services	Non-RCT & unmatched similar service & participants
Bejerholm et al. (2017)	Moderate to severe mental health	Affective disorders (depressive episode, recurrent depression, bipolar disorder, mania or hypomania)	Sweden	33	25	IPS + motivational interviewing + cognitive strategies + structured time-use patterning	Good	Non-integrated BAU services	RCT
Brinchmann et al. (2024)	Common mental health or somatic disorder	Adults 18–40 years old with significantly reduced capacity to work due to medical condition(s), receiving the work assessment allowance benefit and mental health support	Norway	561	3150	IPS	Fair to good	Non-integrated BAU	Difference-in-difference estimation
LePage et al. (2016)	Substance misuse and/or common mental health	Formerly incarcerated veterans with substance issues (88 %) and/or mental health issue (59 %) of which predominantly depression	US	46	38	Modified IPS (no integration, larger caseloads, some exclusions, some mandatory components)	Fair	Non-integrated BAU services	RCT
Lones et al. (2017)	Substance misuse	Opioid misuse	US	22	23	IPS	Fair	Non-integrated BAU services	RCT waitlist control group
Marsden et al. (2024)	Substance misuse	Opioid, alcohol, cannabis and stimulants	UK	844	843	IPS	Fair to good	Non-integrated BAU services	RCT
Rosenheck and Mares (2007)	Homeless veterans and with substance misuse and/or moderate to severe mental health	82 % present with alcohol or drug abuse or dependence. Of mental health conditions present the largest groups are major affective disorder (36 %) and personality disorder (31 %)	US	321	308	Modified IPS (limited integration, no staffing items)	Fair to good. One weaker fidelity sites.	Non-integrated BAU services	Multi-site pre-post nonequiv-alent control group design
Ottomanelli et al. (2017)	Spinal cord injury	Veterans with spinal cord injury (with dominant co-morbidities being 32 % also presenting with hypertension, 35 % with depression, 29 % with substance issues)	US	81	76	IPS	Fair	Non-integrated BAU services	RCT via biased coin randomiz-ation
Sveinsdottir et al. (2020)	NEET young adults	18–29 yr olds not in employment education or training, in receipt of benefits, subject to work activation expectations and wanting to move into competitive employment.	Norway	46	37	IPS (no integration)	Below fair improved to fair.	Traineeship in a sheltered business	RCT
Sveinsdottir et al. (2022)	Chronic pain	Workless Oslo residents eligible for interdisciplinary hospital treatment.	Norway	38	20	IPS	Fair	Inter-disciplinary pain treatment only.	Unbalanced RCT

### 3.4 Risk of bias

Table 3 shows the risk of bias assessment for the 18 studies using the revised Cochrane tool. Two studies show weak counterfactual designs, several studies deviate from the IPS intervention model, and one study does not report the primary job entry outcome. Otherwise there is low risk of bias across studies. Overall risk of bias is low.

**Table 3:** Risk of bias assessment.

**Study**	**Bias due to randomisation process**	**Bias due to deviation from intended intervention**	**Bias due to missing outcome data**	**Bias due to outcome measurement**	**Bias due to selection of the reported result**	**Overall risk of bias**
Reme et al. (2015)	+	?	+	+	+	+
Hellstrom et al. (2017)	+	?	+	+	+	+
Davis et al. (2022)	+	+	+	+	+	+
Newton et al. (2023)	+	+	+	+	+	+
Davis et al. (2012)	+	+	+	+	+	+
Davis et al. (2018)	+	+	+	+	+	+
Reme et al. (2019)	+	+	+	+	+	+
Poremski et al. (2017)	+	?	+	+	+	+
Ferguson et al. (2012)	X	+	+	+	+	?
Bejerholm et al. (2017)	+	?	+	+	+	+
Brinchmann et al. (2024)	?	+	X	+	+	?
LePage et al. (2016)	+	?	+	+	+	+
Lones et al. (2017)	+	+	+	+	+	+
Marsden et al. (2024)	+	+	+	+	+	+
Rosenheck and Mares (2007)	X	?	+	+	+	?
Ottomanelli et al. (2017)	+	+	+	+	+	+
Sveinsdottir et al. (2020)	+	?	+	+	+	+
Sveinsdottir et al. (2022)	+	+	+	+	+	+

Key: + = low risk/no or few concerns;? = medium risk/some concerns; X  = high risk/substantial concern.

Despite fidelity being central to IPS delivery and its evidenced effectiveness (Winter et al., 2020, Yamaguchi et al., 2022) fidelity was not reported in three IPS studies (Hellstrom et al., 2017, Reme et al., 2015, Ferguson et al., 2012). A common finding is that fidelity is neither static nor naturally occurring but instead tends to start low and improve with time. Despite these improvements fidelity rarely reached excellent and frequently did not reach good levels within the study timeframes.

### 3.5 Summary and synthesis of results

**Fig. 2:**
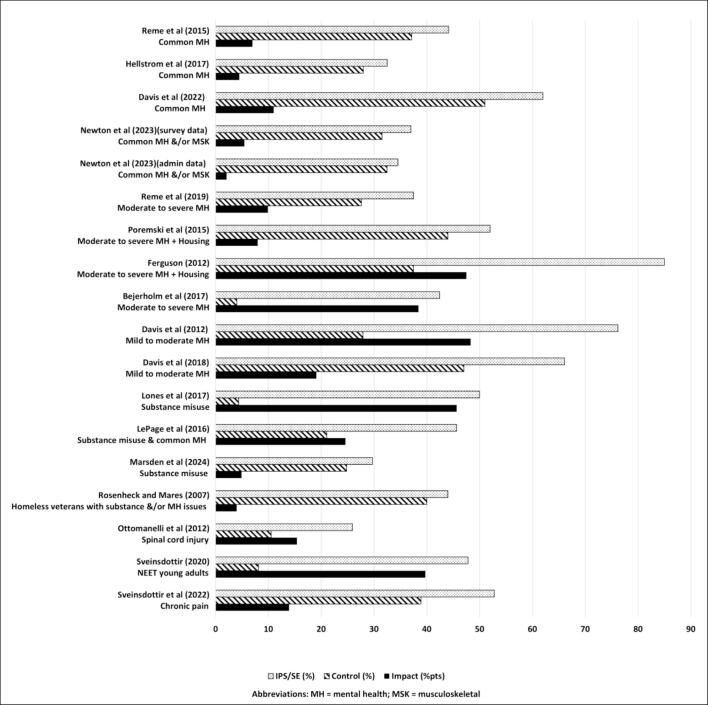
Job entry rates across the individual studies.

The primary vocational outcome of any employment support programme is the job entry rate, by which is meant the percentage of initially workless programme participants who move into paid employment. Fig. 2 presents the job entry rates for IPS participants (dotted bars) and control groups (diagonally shaded bars). Also presented are impact estimates (solid black bars) calculated as the percentage point difference between them. One study only reported outcomes in terms of additional work days and is therefore not able to be included in Fig. 2 (Brinchmann et al., 2024). All studies show positive impact estimates with a range of effects from 4 percentage points up to nearly 50 percentage points. Five studies show very large impacts of around or above 40 percentage points, six studies show large effects of between 10–25 percentage points, and seven studies show more modest impacts of around or below 10 percentage points. The job entry rates themselves are typically large for these population groups: they vary from a low of around 25 percent up to over 70 percent in two studies and around or above 40 percent in twelve studies. For Reme et al. (2019) Fig. 2 shows the job entry rate for participants with common mental health only. For Newton et al. (2023) it displays findings from both the participant survey and the administrative data given the large size of this recent trial and the slight difference between them.

The definition of job entry varies across studies. In most cases the measure relates to the percentage of participants who achieve a job start at any time *within* a specified number of months from their start on the IPS programme. The duration of the follow-up period varies considerably however: 6 months (LePage et al., 2016 67., Lones et al., 2017) or 8 months (Poremski et al., 2017) for several studies; within 12 months is most common (Sveinsdottir et al., 2022, Newton et al., 2023, Sveinsdottir et al., 2020, Davis et al., 2012, Ottomonalli et al., 2012, Davis et al., 2018); and within 24 months for one study (Rosenheck and Mares, 2007). One study measures paid work of greater than one week duration within a 12 month follow-up period (Davis et al., 2022). Conversely, five studies measure whether participants are in paid employment *at* a particular point in time with that period being 12 months after the participant’s start on programme in 4 studies (Hellstrom et al., 2017, Reme et al., 2015, Bejerholm et al., 2017, Reme et al., 2019). The definition of what constitutes a successful job start is not reported in any study and our assumption is that it relates to paid employment of any length. Further agreement on core definitions and reporting measures would be helpful across the IPS research and policy community to aid future comparability.

**Fig. 3:**
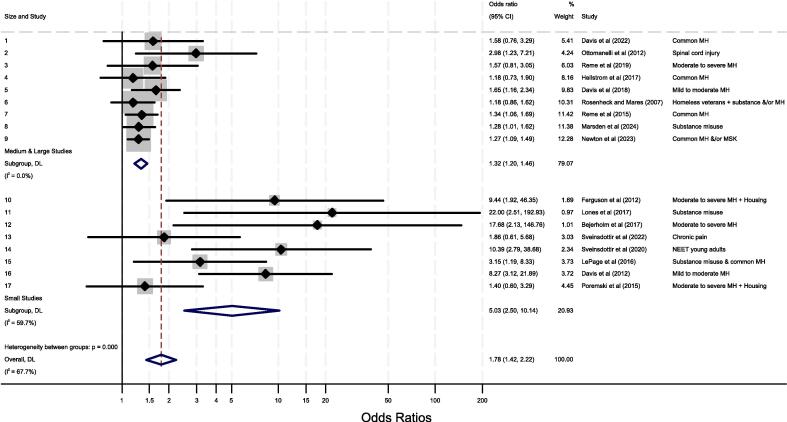
Meta-analysis of IPS impacts across the studies.

Fig. 3 turns to the meta-analysis of overall effects of the primary job entry outcomes. An initial pooled meta-analysis shown in Fig. C1 of the online supplementary material estimated an overall effect of 1.78 [1.42,2.22] but also identified substantial heterogeneity (I^2^ = 68 %). This is in line with previous review evidence (Bond et al., 2019). There is a strong suggestion of small-study effects and potential publication bias. In response, our analyses examine the nature and impact of that heterogeneity. Meta-analysis was repeated with the studies split into three groups according to study total sample size: below 100 (small studies); between 100 and 1000 (medium sized studies); and greater than 1000 (large studies). Fig. C2 in the online supplementary material shows the results with heterogeneity now high only amongst the small studies group (I^2^ = 60 %) but low amongst both medium sized (I^2^ = 9 %) and large (I^2^ = 0 %) studies. Sub-group forest plots are ordered by study sample size (largest at the bottom) within each group. As a next step medium and large sized studies were therefore combined whilst small studies remain grouped together. This seems the optimal grouping for the meta-analysis and is presented in Fig. 3. Amongst large and medium sized studies combined the overall effect is 1.32 [1.20,1.46) and heterogeneity is low (I^2^ = 0 %). Amongst smaller studies the overall effect is 5.03 [2.50,10.14] and heterogeneity is high (I^2^ = 60 %). Six studies show statistically insignificant effects at the 95 % level. For information, similar sub-group meta-analyses were explored across health conditions and geographical region but did not show similar discriminatory power. Their forest plots are shown in the online supplementary materials Figs. C3 and C4 respectively.

To explore heterogeneity further contour funnel plots with the random-effect model were used. Fig. D1 in the supplementary online materials shows a counter funnel plot for all studies and displays clear asymmetry. All of the smaller studies show larger effect sizes and tend to be statistically significant whilst the larger and more precise studies are smaller and more tightly clustered in their effect sizes. However, since the ‘missing’ studies that would be needed to make the funnel symmetric lie primarily in the non-significant dark grey area of the plot then this suggests that the observed asymmetry is not due to publication bias. For policy makers interested in the likely effects of scaled-up IPS interventions in different population groups the overall effect of 1.32 [1.2,1.46] estimated from the large and medium sized studies combined may therefore be a more appropriate measure of the underlying IPS effect at realistic intervention scale than the all study overall estimate of 1.78 [1.42,2.22].

Individual studies also present a range of secondary vocational outcomes beyond the primary outcome measure of job entry rates, although these are frequently reported only in individual studies and not in existing reviews. Table 4 details these findings by study alongside definitions of the measures. For IPS and control groups in turn, Table 4 shows the sample sizes that the measures are based on, mean value (or percentage value if appropriate for that measure), standard deviation and the p-value for the difference in means between IPS and control groups. To aid interpretation the final column presents a ratio of the IPS and control group mean (or percentage): values greater than 1 denote superior IPS performance compared to its control group, with the exception of the time to job entry/return to work measures where smaller values represent shorter/stronger performance. Two of the studies (Reme et al., 2015, Ottomonalli et al., 2012) listed in Table 2 have separate follow-on studies (Overland et al., 2018, Ottomanelli et al., 2017) and these are included in Table 4 in italics.

**Table 4:** Secondary vocational outcomes evidence.

			**Supported Employment**			**Control**				
**Study**	**Population**	**Definition**	***n***	**Mean**/**%**	**SD**	***n***	**Mean**/**%**	**SD**	**Sig**	**Ratio**
***Total work hours***										
LePage et al. (2016)	Substance misuse and/or CMH	Total work hours over 6 month follow-up if employed	46	266	254	38	217	176	ns	1.23
Bejerholm et al. (2017)	Affective disorders	Total work hours within 12 m follow-up (all participants)	33	210.4	432.8	25	3.84	19.2	0.01	54.79
Davis et al. (2012)	Mild to moderate mental health	Total work hours within 12 m follow-up (all participants)	42	656	661	43	236	494	0.00	2.78
Sveinsdottir et al. (2020)	NEET young adults	Total work hours within 12 m follow-up (all participants)	43	140.0	249.4	37	13.95	55.48	0.00	10.04
Marsden et al. (2024)	Substance misuse	Total days employed within 18 m follow-up if employed	207	132.6	141.6	175	130.4	137.3	ns	1.02
Brinchmann et al. (2024)	CMH or somatic disorder	Average additional work days due to IPS per year per person	561	5.6	−	3150	0	−	0.00	−

***Weekly hours worked***										
LePage et al. (2016)	Substance misuse	Weekly work hours over 6 month follow-up if employed	46	36	24	38	33	22	ns	1.09
Bejerholm et al. (2017)	Affective disorders	Weekly hours at 12 m follow-up (all participants)	33	11.0	17.3	25	0.3	1.6	0.00	36.67
Sveinsdottir et al. (2020)	NEET young adults	Percentage ever working ≥20 h per week during 12 m follow-up (all participants)	42	33.3	−	37	5.4	−	0.00	6.17
Ottomanelli et al. (2017)	Spinal cord injury	Weekly hours during 12 m follow-up if employed	24	22.0	14.6	9	17.0	14.6	<0.05	1.29
Poremski et al. (2017)	Moderate to severe MH	Weekly hours if employed	23	38.7	−	18	23.2	−	0.10	1.67
Ferguson et al. (2012)	Moderate to severe mental health + housing issues	Weekly hours during the 10 month follow-up (all participants)	16	33.4	3.95	20	32.5	10.6	ns	1.03
Sveinsdottir et al. (2020)	Chronic pain	Percentage ever working ≥20 h per week during 12 m follow-up (all participants)	38	17.6	−	20	11.8	−	0.70	1.49
Ottomanelli et al. (2017)	*Spinal cord injury*	Weekly hours worked if employed	17	19.3	16.2	6	22.1	25.4	−	0.87

***Job sustainment***										
LePage et al. (2016)	Substance misuse	Days worked within 6 month follow-up if employed	46	88.3	53.0	38	87.6	50.0	ns	1.01
Bejerholm et al. (2017)	Affective disorders	Weeks worked within 12 m follow-up (all participants)	33	7.7	13.4	25	0.6	2.5	0.01	12.83
Davis et al. (2012)	Veterans with PTSD	Total work days within 12 m follow-up (all participants)	42	83.8	80.6	43	29.3	61.9	0.00	2.86
Davis et al. (2012)	Veterans with PTSD	Total work weeks within 12 m follow-up (all participants)	42	21.6	17.1	43	6.8	13.8	0.00	3.18
Ottomanelli et al. (2017)	Spinal cord injury	Employment duration if employed (weeks)	24	17.3	13.1	9	24.8	16.0	<0.05	0.70
Poremski et al. (2017)	Moderate to severe MH	Employment duration if employed (days)	23	58	−	18	79	−	0.46	0.73
Hellstrom et al. (2017)	Common mental health	Weeks worked within 12 m follow-up (all participants)	162	11.6	1.35	164	9.9	1.34	0.38	1.17
Hellstrom et al. (2017)	Common mental health	Weeks worked within 24 m follow-up (all participants)	162	32.4	2.76	164	26.7	2.74	0.22	1.21
Davis et al. (2022)	Common mental health	Days in paid work during 12 m follow-up if employed	36	177.0	61.9	31	123.3	82.8	0.00	1.44
Davis et al. (2022)	Common mental health	Holding paid work for ≥6m of the 12 m follow-up	58	45.0	−	61	25.0	−	0.02	1.80
Newton et al. (2023)	Common MH &/or physical health	Weeks in paid employment during 12 m follow-up	1510	7.3	0.5	1380	7.1	0.5	0.77	1.03
Ferguson et al. (2012)	Moderate to severe mental health + housing issues	Months worked during the 10 month follow-up (all participants)	16	5.2	3.3	20	2.2	3.0	0.01	2.36
Davis et al. (2018)	Mild to moderate mental health	Weeks employed during 18 month follow-up (all participants)	271	17.5	17.7	270	12.1	15.5	0.00	1.45
Davis et al. (2018)	Mild to moderate mental health	Days employed during 18 month follow-up (all participants)	271	122.3	124.2	270	84.9	108.1	0.00	1.44
Lones et al. (2017)	Substance misuse	Days worked during 12 month follow-up (if employed)	11	177	−	5	156	−	−	1.13

			**Supported Employment**			**Control**				

**Study**	**Population**	**Definition**	***n***	**Mean**/**%**	**SD**	***n***	**Mean**/**%**	**SD**	**p-value**	**Ratio**

Rosenheck and Mares (2007)	Homeless veterans with substance misuse &/or moderate/severe MH	Days worked per month (all participants)	321	8.8	−	308	5.6	−	−	1.57
Rosenheck and Mares (2007)	Homeless veterans with substance misuse &/or moderate/severe MH	Days worked during 24 month follow-up (all participants)	321	34.1	−	308	29.8	−	0.04	1.14
Sveinsdottir et al. (2022)	Chronic pain	Hours worked during 12 month follow-up (all participants)	38	216.5	447.3	20	122.9	255.2	0.43	1.76
Marsden et al. (2024)	Substance misuse	Longest employment spell within 18m follow-up if employed	207	110.2	120.9	175	114.2	126.0	ns	0.96
Marsden et al. (2024)	Substance misuse	Employed 13+ weeks in 18m follow-up if employed	207	87=42%	−	175	73=42%	41.7	ns	1.01
Overland et al. (2018)	*CMH*	*Employment no benefit for at least 24 of 36 months follow-up*	*630*	*38.8*	*−*	*563*	*37.0*	*−*	*0.04*	1.05
Ottomanelli et al. (2017)	*Spinal cord injury*	*Weeks in paid employment of 24 month follow-up if employed*	*17*	*22.2*	*14.2*	*6*	*18.7*	*13.5*	*−*	1.19

***Total income earned***										
LePage et al. (2016)	Substance misuse	Total wages over 6 month follow-up if employed ($)	46	2,761	2,697	38	2,866	2,571	ns	0.96
Bejerholm et al. (2017)	Affective disorders	Net income at 12m follow-up (Euros) (all participants)	54	1,565	−	54	1,048	−	0.00	1.49
Davis et al. (2012)	Veterans with PTSD	Total earned income in 12m follow-up ($) (all participants)	42	9,264	13,294	43	2,601	6,009	0.00	3.56
Ottomanelli et al. (2017)	Spinal cord injury	Weekly wages ($) if employed	24	233.9	279.0	9	267.3	462.5	<0.05	0.88
Davis et al. (2022)	Common mental health	Earned income during 12m follow-up ($) if employed	36	18,945	10,792	31	13,813	10,809	0.06	1.37
Newton et al. (2023)	Mental &/or physical health	Earnings in month 12 post-randomisation (£)	3,636	214	11	3,630	214	11	1.00	1.0
Ferguson et al. (2012)	Moderate to severe mental health + housing issues	Weekly earned income during the 10 month follow-up ($)(all participants)	16	263.6	147.6	20	192.5	116.7	ns	1.37
Davis et al. (2018)	Mild to moderate mental health	Earned income during 18 month follow-up($)(all participants)	271	14,642	19,308	270	10,989	17,097	0.00	1.33
Ottomanelli et al. (2017)	*Spinal cord injury*	*Earned wages per week in 24 month follow-up if employed ($)*	*17*	*251.0*	*276.0*	*6*	*70.4*	*110.9*	*−*	3.57

***Hourly wage if employed***										
LePage et al. (2016)	Substance misuse	Hourly wage over 6 month follow-up if employed ($)	46	10.38	−	38	13.21	−	ns	0.79
Poremski et al. (2017)	Moderate to severe mental health	Hourly wage if employed ($)	23	16.82	−	18	13.19	−	0.34	1.28
Lones et al. (2017)	Substance misuse	Hourly wage if employed ($)	11	12.84	−	5	13.25	−	−	0.97
Rosenheck and Mares (2007)	Homeless veterans with substance misuse &/or moderate/severe MH	Hourly wage if employed ($)	321	8.52	−	308	8.10	−	−	1.05

***Time to job entry/return to work***										
LePage et al. (2016)	Substance misuse	Days to job entry/return to work	46	130.7	63.6	38	157.1	50.1	0.02	0.83
Davis et al. (2012)	Veterans with PTSD	Weeks to job entry (all participants)	Survival curve shows notably faster time to job entry of IPS group compared to control but no statistics are reported							
Davis et al. (2022)	Common mental health	Weeks to job entry	58	8.7	8.8	61	17.1	14.2	0.00	0.51
Davis et al. (2018)	Mild to moderate mental health	Weeks to job entry	271	18.4	15.1	270	28.2	20.0	0.00	0.65
Marsden et al. (2024)	Substance misuse	Time to first job if employed	207	179.7	142.6	175	201.1	151.9	ns	0.89

***Job search self-efficacy***										
Newton et al. (2023)	CMH &/or physical health		1445	3.2	0.0	1317	3.1	0.0	0.01	1.03

***Job classification***										
Davis et al. (2018)	Mild to moderate mental health	Hollingshead job classification % in occupational categories (1,2,3/4,5/6/7)	276	20/45/55/29	−	211	19/50/37/31	−	ns	

Table 4 shows that performance across this range of wider vocational outcomes is generally stronger in IPS services than control groups. In terms of total work hours all studies show superior IPS performance compared to controls and with some large differences evident. For weekly hours worked all but one study shows superior IPS performance. Most positive results are between ratios of 1 and 1.6 but with two studies showing markedly larger weekly hours for IPS compared to their controls. In terms of the average weekly hours worked these are typically – though not always (Sveinsdottir et al., 2020) – long part-time or towards full-time weekly hours. Job sustainment is a key measure of the performance of any employment programme. Job sustainment is stronger in IPS services than control groups in all but three studies with most ratios being between 1 and 2 and a small number larger than 2. Most studies show larger total earnings from employment in IPS services than in control groups with most of those showing improvements of between around a third and fifty percent over control group earnings. Two studies report earnings amongst IPS participants of over 3.5 times that of control group participants. In terms of hourly wage the evidence is more mixed with ratios that are close to one, modestly negative and modestly positive. The consistently positive results around total earnings in IPS services therefore seem driven by superior performance in weekly hours and job sustainment rather than by improvements in hourly wages. Five studies report time in the employment service until job entry, although one reports graphically only (Davis et al., 2012). All studies report faster average time to job start in IPS services compared with control groups with three studies suggesting return to work occurs around or towards twice as quickly in IPS services compared to control groups (Davis et al., 2022, Davis et al., 2012, Davis et al., 2018) and the two other studies suggest 10 %-20 % faster (Marsden et al., 2024, LePage et al., 2016 67.). One study reports on impacts on job search self-efficacy and finds a small positive impact in favour of IPS (Newton et al., 2023). Finally, one study reports minimal evidence of differences in the occupation categories of job starts between IPS and control (Davis et al., 2018).

### 3.6 Reporting biases

Reporting bias is low. There is confidence that relevant studies were identified, data extraction was exhaustive at both review and study level and there was minimal missing data.

### 3.7 Certainty of evidence

Following the GRADE criteria there is moderate certainty in the findings. Overall risk of bias is low. Precision is moderately high: the overall effect is of large magnitude and always positive, though with large uncertainty. Precision is higher when focusing only on the overall effect estimated from the large and medium sized studies combined. Studies show consistently positive effects but effects vary in size, the measurement of outcome variables varies across studies, and many samples are of moderate size. Regards indirectness, studies cover a range of population groups although are concentred amongst mental health and, to a lesser extent, substance misuse and relate to limited range of advanced economies. There is no evidence of publication bias.

## 4 Discussion

This overview review provides a consolidated overview review and meta-analysis of the rapidly evolving research and policy landscape around IPS employment interventions for population groups other than severe mental health.

We identify 5 eligible reviews and 18 individual studies for inclusion. Studies show consistent positive evidence for the superior performance of IPS approaches compared to business-as-usual across most outcomes including job entry, average time taken to move into employment, job sustainment, work hours and total earnings. More mixed evidence was found in relation to average hourly wage. Evidence is limited and weak regards job search self-efficacy and occupational classification. Substantial heterogeneity was identified by study size and the overall weighted odds ratio of 1.32 [1.2,1.46] estimated from the large and medium sized studies seems a more plausible estimate of the likely effects of scaled-up IPS interventions in groups beyond severe mental health. Although positive results are seen across all population groups it is notable that these studies are concentred amongst mental health (albeit at low to moderate levels and often in conjunction with other conditions) where IPS is already well evidenced to be effective for severe mental health groups. Overall risk of bias is low and there is moderate certainty in the evidence.

The review is limited by inconsistent and vague terminology in the literature that may have prevented some studies being identified and population groups or intervention types being difficult to clearly identify. The evidence base displays data limitations including frequently small sample sizes, inconsistency regards the definition of vocational outcome measures, inconsistent reporting, and variation in and uncertainty of control group interventions. Additionally, there are limitations in several studies regards contamination of IPS with other interventions and non-adherence to fidelity. A pervasive limitation across the studies is rapid evaluation timelines such that fidelity remains immature and still developing. One author (MC) conducted the search strategy and one author (AW) conducted risk and bias and certainty assessments. We do not expect this to have impacted the findings.

It is interesting to step back and place these findings in the context of international IPS scholarship and practice. One recent review of IPS in its traditional severe mental health population groups summarises a range of job entry risk ratios across studies in the range 1.54–2.49 (Drake and Bond, 2023), somewhat higher than the equivalent 1.19 risk ratio seen in the large and medium studies or 1.40 risk ratio seen across all studies of IPS beyond severe mental health outlined above in the present overview review. Nevertheless, consistent positive findings in favour of IPS across diverse population groups within and beyond severe mental health are now evident. Beyond severe mental health, these findings highlight that although IPS has been shown with statistical significance to be effective in relation to substance misuse, NEET young, spinal cord injury, PTSD, affective disorder and musculoskeletal conditions the evidence remains concentrated amongst (low to moderate) mental health conditions, whether alone or in combination with other conditions. Two key messages for future policy and research emerge. Firstly, the evidence suggests that whilst IPS can be effective beyond mental health that further trialling is warranted in these wider population groups beyond mental health. Second, it would be beneficial if studies began to routinely report itemised fidelity scores so that we may begin to better understand the associations between individual model elements and the consistently positive effect sizes being seen. Doing so will help us to assess how best to target and maximise the fit and effectiveness of IPS interventions across different population groups in the future.

## 5 Conclusions

This overview review offers the most comprehensive, consistent and current overview review and meta-analysis of international evidence of the impact on vocational outcomes of IPS employment interventions in population groups other than severe mental health. Health-related worklessness is a significant policy challenge across all advanced economies and policy makers across nations are experimenting increasingly with IPS in diverse population groups as they seek more effective policy solutions. These findings offer support for the continued policy focus on IPS experimentation and expansion as an evidence-based approach to health-related worklessness in a wide variety of different population groups and settings. Findings also highlight key limitations and priorities to address in future policy and research.

## 6 Registration and protocol

Registered with the PROSPERO database number [blinded for review]. A protocol was not prepared.

## 7 Statement of ethical approval

The study was granted ethical approval by the University of Strathclyde.

## 8 Declaration of Generative AI and AI-assisted technologies in the writing process

No generative AI or AI-assisted technologies were used in the writing process.

## CRediT authorship contribution statement

**Adam Whitworth:** Writing – review & editing, Writing – original draft, Visualization, Supervision, Resources, Project administration, Investigation, Funding acquisition, Formal analysis, Data curation, Conceptualization. **Susan Baxter:** Writing – review & editing, Validation, Supervision, Software, Resources, Methodology, Investigation, Data curation, Conceptualization. **Jane Cullingworth:** Writing – review & editing, Investigation, Data curation. **Mark Clowes:** Investigation.

## Declaration of competing interest

The authors declare that they have no known competing financial interests or personal relationships that could have appeared to influence the work reported in this paper.

## Data Availability

No data was used for the research described in the article.
