# Supplementary material for: Individual Placement and Support (IPS) beyond severe mental health: An overview review and meta-analysis of evidence around vocational outcomes
Source: Prev Med Rep. 2024 Jun 10;43:102786. doi: 10.1016/j.pmedr.2024.102786 (PMC11225006; doi:10.1016/j.pmedr.2024.102786)
Supplement: Supplementary Data 1 [file mmc1.docx]

**Supplementary materials A: Search keywords and example search strategy**

***Search Keywords:***

individual placement and support; IPS and placement* and (work* or job* or employ* or unemploy*); employment, supported; support* or placement* and welfare-to-work; work* or job* or employ*; supported employment

***Example search strategy:***

Ovid MEDLINE(R) ALL 
1 "individual placement and support".mp. 
2 (IPS and placement* and (work* or job* or employ* or unemploy*)).mp. 
3 ("individual* placement*" and support*).mp. 
4 Employment, Supported/ 
5 ((support* or placement*) and (welfare-to-work or (ex-offender* and (work* or job* or employ*)))).mp. 
6 "supported employment".mp. 
7 1 or 2 or 3 or 4 or 5 or 6 
8 limit 7 to (english language and yr="2000 -Current") 
9 exp Schizophrenia/ or schizophreni*.mp. 
10 (Bipolar Disorder/ or [bipolar.mp](http://bipolar.mp/). or [bi-polar.mp](http://bi-polar.mp/).) and [severe.mp](http://severe.mp/). 
11 ((psychiatr* and severe) or (psychos* or psychot*)).mp. 
12 ((psychol* or depress* or anxi* or (mental* adj2 (ill* or health))) and severe).mp. 
13 9 or 10 or 11 or 12 
14 8 not 13  *(i.e. probably not SMI)*
15 8 and 13  *(i.e. probably SMI)*

**Supplementary materials B: Studies excluded from the reviews and rationale**

| **Individual study excluded from this overview review** | **Rationale for exclusion** | **Review study in which individual study is located** |
| --- | --- | --- |
| Meuser et al (2011) | Severe mental illness | Harrison et al (2020) |
| Cook et al (2007) | Not SE to fidelity |  |
| Beimers et al (2010) | No robust counterfactual |  |
| Frounfelker et al (2011) | No robust counterfactual |  |
| Bond et al (2015) | Severe mental illness | Probyn et al (2021) |
| Li-Tsang et al (2008) | Not SE to fidelity |  |
| Magura et al (2007) | Not SE to fidelity |  |
| Killackey et al (2008) | Severe mental illness | Jetha et al (2019) |
| Baksheev et al (2012) | Severe mental illness |  |
| Burke-Miller et al (2012) | Severe mental illness |  |
| Cook et al (2005) | Severe mental illness |  |
| Fraker et al (2018) | Not SE to fidelity |  |
| Major et al (2010) | Severe mental illness |  |
| McGahey et al (2014) | Severe mental illness |  |
| Smith et al (2015) | Not SE to fidelity |  |
| Wehman et al (2017) | Not SE to fidelity |  |
| Yamaguchi et al (2016) | Severe mental illness |  |

**Supplementary materials C: Meta-analysis sub-group analyses**

***Supplementary Figure C1: Pooled meta-analysis forest plot***

***Supplementary Figure C2: Meta-analysis forest plot by three grouped study size***

******

***Supplementary Figure C3: Meta-analysis forest plot by health condition groups***

******

***Supplementary Figure C4: Meta-analysis forest plot by geography***

******

**Supplementary online material D: Funnel plots and trim-and-fill analyses**

***Supplementary Figure D1: Counter-enhanced funnel plot of all studies***

**
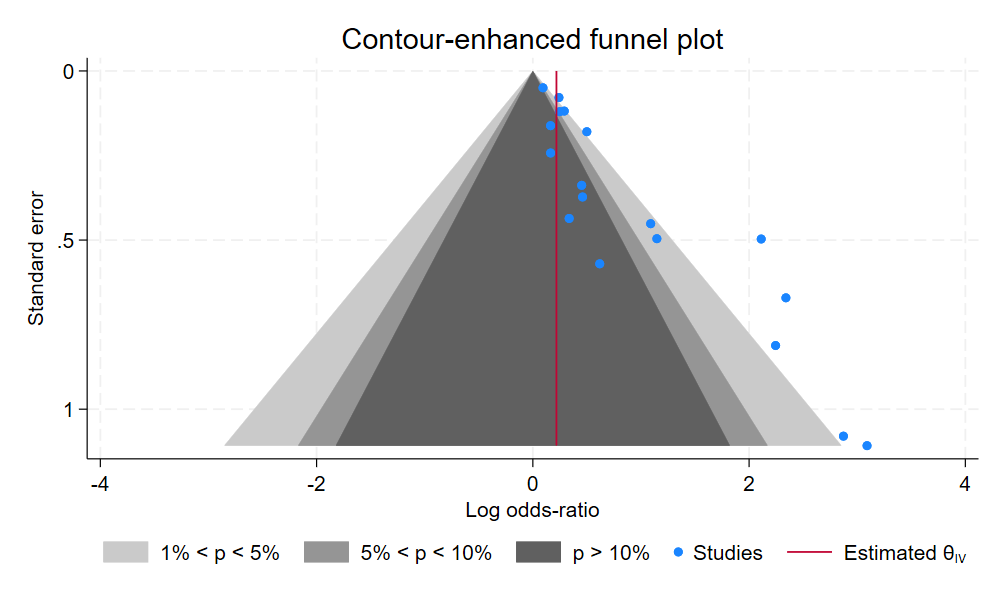
**
